# Untangling the Effect of Carbonaceous Materials on the Photoelectrochemical Performance of BaTaO_2_N

**DOI:** 10.1021/acsomega.3c08894

**Published:** 2024-01-29

**Authors:** Mirabbos Hojamberdiev, Ronald Vargas, Lorean Madriz, Zukhra C. Kadirova, Kunio Yubuta, Fuxiang Zhang, Katsuya Teshima, Martin Lerch

**Affiliations:** † Institut für Chemie, Technische Universität Berlin, Straße des 17. Juni 135, 10623 Berlin, Germany; ‡ Instituto Tecnológico de Chascomús (INTECH), Consejo Nacional de Investigaciones Científicas y Técnicas (CONICET), Universidad Nacional de San Martín (UNSAM), Avenida Intendente Marino, Km 8,2, B7130IWA Chascomús, Provincia de Buenos Aires, Argentina; § Escuela de Bio y Nanotecnologías, Universidad Nacional de San Martín (UNSAM), Avenida Intendente Marino, Km 8,2, B7130IWA Chascomús, Provincia de Buenos Aires, Argentina; ∥ Uzbekistan–Japan Innovation Center of Youth, University Street 2B, 100095 Tashkent, Uzbekistan; ⊥ Department of Applied Quantum Physics and Nuclear Engineering, Kyushu University, Fukuoka 819-0395, Japan; # State Key Laboratory of Catalysis, Dalian National Laboratory for Clean Energy, iChEM, Dalian Institute of Chemical Physics, Chinese Academy of Sciences, Dalian 116023, China; ∇ Department of Materials Chemistry, Shinshu University, 4-17-1 Wakasato, Nagano 380-8553, Japan; ○ Research Initiative for Supra-Materials, Shinshu University, 4-17-1 Wakasato, Nagano 380-8553, Japan

## Abstract

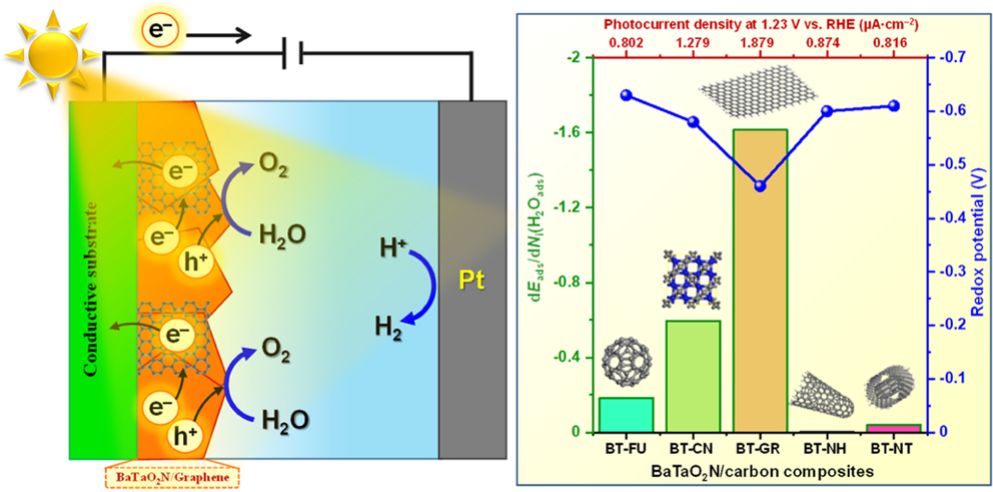

The water oxidation reaction is a rate-determining step in solar water splitting. The number of surviving photoexcited holes is one of the most influencing factors affecting the photoelectrochemical water oxidation efficiency of photocatalysts. The solar-to-hydrogen energy conversion efficiency of BaTaO_2_N is still far below the benchmark efficiency set for practical applications, notwithstanding its potential as a 600 nm-class photocatalyst in solar water splitting. To improve its efficiency in photoelectrochemical water splitting, this study offers a straightforward route to develop photocatalytic materials based on the combination of BaTaO_2_N and carbonaceous materials with different dimensions. The impact of diverse carbonaceous materials, such as fullerene, g-C_3_N_4_, graphene, carbon nanohorns, and carbon nanotubes, on the photoelectrochemical behavior of BaTaO_2_N has been examined. Notably, the use of graphene and g-C_3_N_4_ remarkably improves the photoelectrochemical performance of the composite photocatalysts through a higher photocurrent and acting as electron reservoirs. Consequently, a marked reduction in recombination rates, even at low overpotentials, leads to a higher accumulation of photoexcited holes, resulting in 2.6- and 1.7-fold increased BaTaO_2_N photocurrent densities using graphene and g-C_3_N_4_, respectively. The observed trends in the dark for the oxygen reduction reaction (ORR) potential align with the increase in the photocurrent density, revealing a good correlation between opposite phenomena. Importantly, the enhancement observed implies an underlying accumulation phenomenon. The verification of this concept lies in the evidence provided by oxygen reduction and is in line with photoredox flux matching during photocatalysis. This research underscores the intricate interplay between carbonaceous materials and oxynitride photocatalysts, offering a strategic approach to enhancing various photocatalytic capabilities.

## 1 Introduction

Photoelectrochemical (PEC) water splitting is one of the potential processes to generate green hydrogen by involving renewable energy.^1,2^ However, the water oxidation reaction on the photoanode requires the transfer of four electrons, in comparison to the two-electron-transfer water reduction reaction on the photocathode. Therefore, the sluggish water oxidation reaction is the rate-determining step that governs the rate of the water-splitting reaction.^3^

BaTaO_2_N is a promising visible-light-active photocatalyst for water oxidation due to its capability to absorb visible light up to 660 nm, appropriate band-edge potential straddling the water oxidation reaction potential, good stability in concentrated alkaline solution, and nontoxicity.^4,405^ Under AM 1.5G simulated sunlight based on an incident photon-to-current conversion efficiency (IPCE) of 100% at <660 nm, the photocurrent density and solar-to-hydrogen (STH) conversion efficiency are assumed to reach approximately 18 mA cm^–2^ and 24%, respectively.^5,6^ To achieve higher efficiency in solar water splitting over BaTaO_2_N, various strategies, such as band-gap engineering via mono- and dual-substitution^7,8^ and solid solutions,^9,10^ controlling the defect density,^11−13^ fabricating thin films,^14,15^ tailoring the exposed surface, morphology, and size,^16−18^ etc., were applied. Photocurrent densities of ∼0.03,^19^ >1.2,^20^ 2.05,^21^ 4.2,^5^ ∼4.5,^22^ and 6.5 mA cm^–2^^23^ at 1.2 V vs the reversible hydrogen electrode were progressively achieved for BaTaO_2_N, while incident photon-to-current efficiencies of 1% at 500 nm,^19^ >4% at 400 nm,^20^ 13% at 420 nm,^21^ ∼30% at 400 nm,^5^ 34–35% at 380–540 nm,^22^ and ≈43% IPCE at 540 nm^23^ at 1.2 V vs RHE steadily increased. Even though the half-cell solar-to-hydrogen energy conversion efficiency of BaTaO_2_N reached 1.4% at 0.88 V_RHE_,^23^ it is still far from the benchmark efficiency set for practical application. Therefore, it is necessary to further explore new ways to improve its efficiency in photoelectrochemical water splitting.

As a straightforward approach, carbon-based nanomaterials have been involved in improving the water-splitting performance of various photocatalysts due to their excellent physicochemical, electrical, mechanical, and optical properties, structural diversity, low cost, and easy synthesis. Carbon-based nanomaterials have been applied as (i) supporting materials for increasing the adsorption sites of active centers and the homogeneous distribution of photocatalyst particles; (ii) cocatalysts for improving the charge separation, reducing the overpotential, providing the catalytic sites, and minimizing the activation energy of hydrogen; (iii) photosensitizers for enhancing the photoresponse of wide-band-gap photocatalysts to visible light with a longer wavelength; and (iv) photocatalysts. The photoanode based on hydrogenated TiO_2_ nanorod arrays decorated with carbon quantum dots exhibited an IPCE value of ∼66.8% and a photocurrent density of ∼3.0 mA cm^–2^ at 1.23 V vs RHE under simulated sunlight, which are 6-fold higher than that of pristine TiO_2_, because the decorated carbon quantum dots acted as electron reservoirs to trap photoexcited electrons and enhanced solar light harvesting due to their upconversion effect.^24^ The ZnFe_2_O_4_ photoanode showed an extremely weak transient photocurrent response, whereas the carbon quantum dot-modified ZnFe_2_O_4_ photoanode exhibited an eight times higher transient photocurrent response because of the efficient separation of photoexcited charge carriers.^25^ The carbon quantum dot-modified Fe_2_O_3_ photoanode demonstrated a 27-fold enhancement in photocurrent density at 0.23 V in comparison to the Fe_2_O_3_-based photoanode owing to the suppression of the recombination of photoexcited charge carriers and enhanced light absorption stemming from the upconversion of carbon quantum dots.^26^ Wang et al.^27^ enhanced the photoelectrochemical water oxidation reaction of the BiVO_4_ photoanode by involving carbon spheres, and the photocurrent density and bulk carrier separation efficiency of carbon sphere-modified BiVO_4_ were significantly higher than that of pristine BiVO_4_ because carbon spheres acted as electron reservoirs, promoting efficient separation of photoexcited charge carriers. The photoelectrochemical performance of hexagonal and monoclinic WO_3_ toward water oxidation under light irradiation was boosted by incorporating them with an amorphous nanoporous carbon additive that facilitated the majority carrier transport through the graphitic layers.^28^

Carbonaceous materials with various dimensions have their own advantages over others and can influence photoelectrochemical performance differently.^29^ In this study, we aim to gain insights into the effect of carbonaceous materials, such as fullerene, g-C_3_N_4_, graphene, carbon nanohorns, and carbon nanotubes, on enhancing the photoelectrochemical performance of BaTaO_2_N for water oxidation. Particularly, the role of carbonaceous materials with various dimensions in the improvement of the light absorption, separation, and transfer of photoexcited charge carriers and photoelectrochemical water oxidation kinetics is discussed here.

## 2 Experimental Section

### 2.1 Synthesis

BaTaO_2_N powders were synthesized by a flux growth method using a localized NH_3_ delivery system^11^ KCl as a flux.^30^ As a solute with a concentration of 10 mol %, BaCO_3_ (99.99%, chemPUR) and Ta_2_O_5_ (99%, Alfa Aeser) were manually mixed in a stoichiometric ratio with KCl (>99.5%, Fluka) as the flux. The well-homogenized mixture was placed in a platinum crucible and heated at 950 °C for 6 h in a horizontal tube furnace with a heating rate of 400 °C h^–1^ and a natural cooling rate under an NH_3_ flow rate of 12.5 L h^–1^. Then, BaTaO_2_N powders were mixed with 20 wt % fullerene (98%, chemPUR), g-C_3_N_4_,^31^ graphene (99.5%, chemPUR), carbon nanohorns (90%, Merck), and carbon nanotubes (90%, Strem Chemicals), and the prepared samples were denoted as BT, BT-FU, BT-CN, BT-GR, BT-NH, and BT-NT.

### 2.2 Characterization

The X-ray diffraction (XRD) patterns were acquired with a PANalytical X’Pert PRO diffractometer with Cu Kα radiation. The microstructures of the samples were examined by scanning electron microscopy (SEM; JSM-7600F, JEOL). The bright-field and lattice images and selected-area electron diffraction (SAED) patterns were observed by transmission electron microscopy (TEM; EM-002B, TOPCON). The ultraviolet–visible (UV–Vis) diffuse reflectance spectra were recorded on an Evolution 220 UV–vis spectrometer (Thermo Fisher Scientific).

### 2.3 Photoelectrochemical Measurements

To determine the photoelectrochemical behavior of the photoanodes, the working electrodes were prepared. First, the BaTaO_2_N/carbonaceous material composites were prepared by mixing the as-synthesized BaTaO_2_N powders with 20 wt % fullerene (98%, chemPUR), 20 wt % g-C_3_N_4_,^31^ 20 wt % graphene (99.5%, chemPUR), 20 wt % carbon nanohorns (90%, Merck), or 20 wt % carbon nanotubes (90%, Strem Chemicals), and then, their corresponding suspensions (1.0 mg mL^–1^) were prepared using an ethanol/water mixture with a 1:1 ratio under ultrasonication for 30 min. The resulting suspensions were evenly deposited onto the Metrohm-DropSens (110) screen-printed electrodes by a dip-coating technique,^32,33^ shielded by a glass beaker, and dried at 80 °C using a heat gun for 10 min. The main contact between BaTaO_2_N particles and carbonaceous materials is expected to be via electrostatics, and connectivity was verified through electrochemical tests. In fact, carbonaceous materials with <20 wt % did not give satisfactory results. Before the photoelectrochemical measurements, cyclic voltammetry (20 mV s^–1^) was conducted on the fabricated electrodes (starting at 0 V vs RHE, with an upper limit of 2.0 V vs RHE and a lower limit of −0.9 V vs RHE) in a deoxygenated supporting electrolyte (0.1 M NaOH), typically running 5 cycles or until a reproducible response was achieved. The photoelectrochemical tests were conducted in a 0.1 M NaOH aqueous solution, which was bubbled with N_2_ for 10 min. The photoelectrochemical measurements were performed by using a light-emitting diode (LED) lamp with a light intensity of ∼100 mW cm^–2^ (Solar Light, G2 V). The counter electrode was a carbon ring concentric to the working electrode, and the reference electrode used was Ag/AgCl. The potential with respect to the Ag/AgCl electrode was converted to that relative to the reversible hydrogen electrode (RHE) using the Nernst relationship^34^
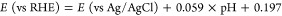
1The photoelectrochemical measurements were performed using a Metrohm-DropSens potentiostat (μSTAT200). The volume of the solution was 100 μL, and the geometric area exposed to light was 0.13 cm^2^. The photoelectrochemical measurements were performed in two modes: (i) potentiodynamic by linear scanning voltammetry (LSV) at 4 mV s^–1^ and (ii) potentiostatic by chronoamperometry (CA) at 1.2 and 1.5 V (vs RHE). The power density (*P*) was calculated according to eq 2
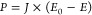
2where *J* is the photocurrent density, *E*_0_ = 1.23 V vs RHE, and *E* is the potential.^35^ In addition, for each fabricated electrode, cyclic voltammetry (CV) was performed under dark conditions at 20 mV s^–1^ in a 0.1 M NaOH aqueous solution with dissolved O_2_ (∼1 mM). The O_2_ concentration was fixed after air bubbling for 5 min at room temperature (25 °C) and measured with the HACH sensor.

## 3 Results and Discussion

### 3.1 Material Characterization

**Figure 1:**
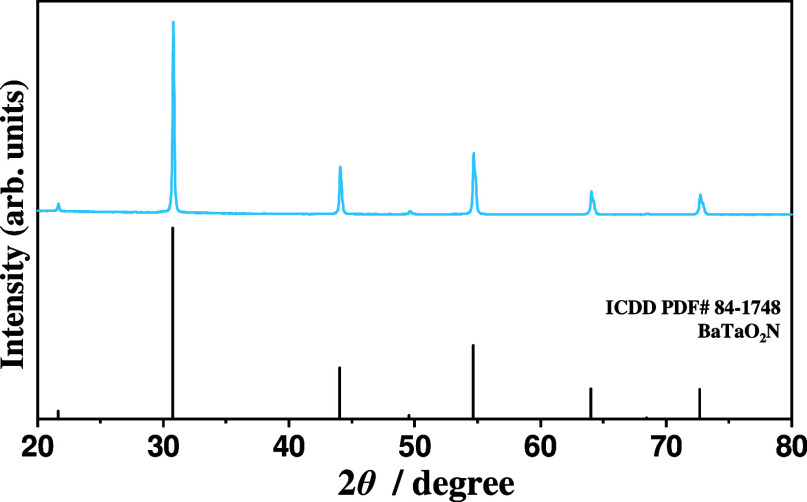
XRD pattern of BaTaO_2_N powders (sample BT).

The synthesized BaTaO_2_N powders were analyzed by X-ray diffraction. Figure 1 shows the XRD pattern of synthesized BaTaO_2_N powders along with an entry from the ICDD-PDF-2 powder pattern database. As shown, all of the intense reflections in the XRD pattern can be readily indexed to the cubic perovskite BaTaO_2_N phase with a space group of *Pm*3̅*m* (No. 221) and unit cell parameters of *a* = *b* = *c* = 4.1128 Å and α = β = γ = 90° (ICDD PDF# 84–1748).^36^ No reflections assignable to the impurity crystalline phase are noted, indicating the high phase purity of the synthesized BaTaO_2_N powders.

**Figure 2:**
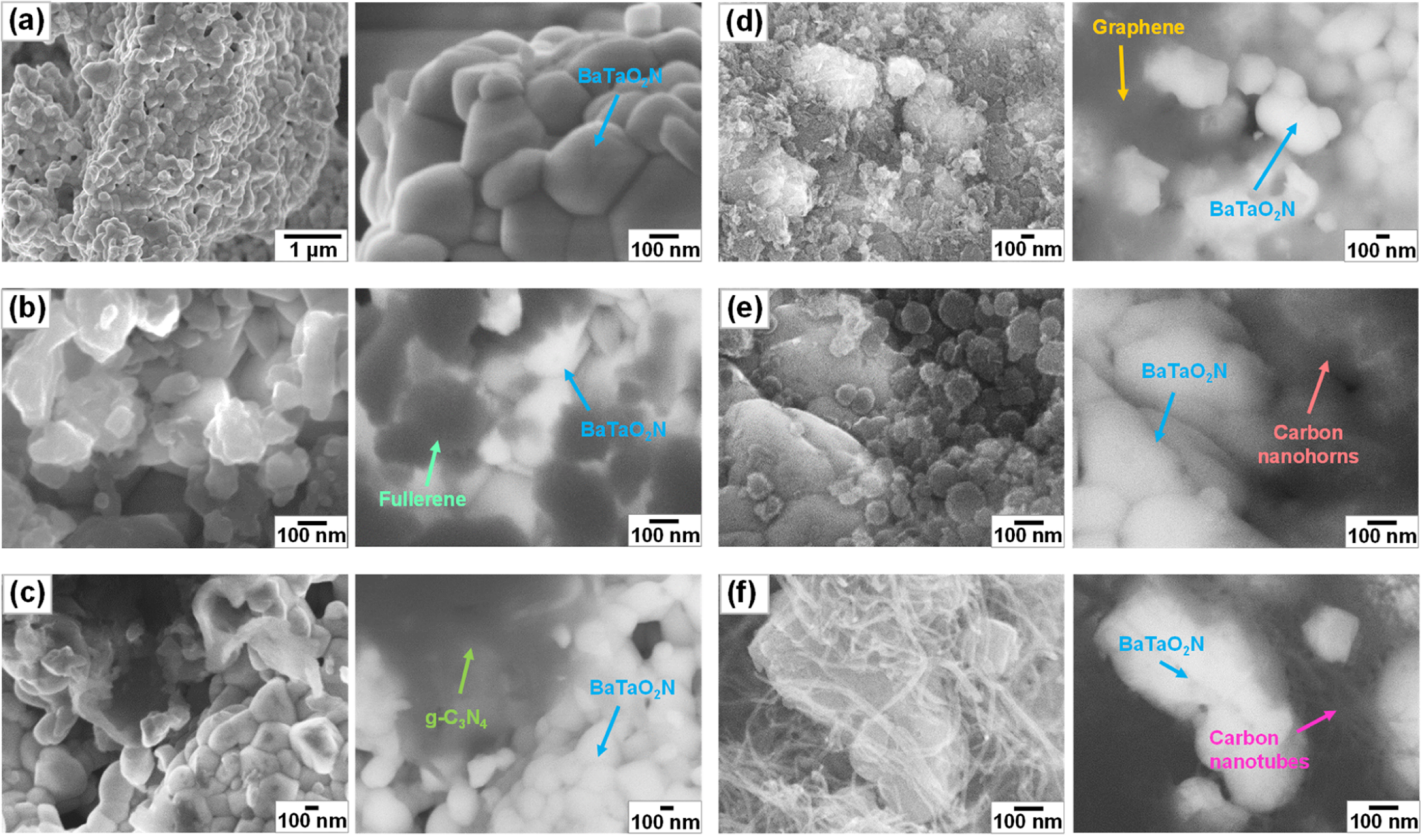
SEM images of BT (a), BT-FU (b), BT-CN (c), BT-GR (d), BT-NH (e), and BT-NT (f).

**Figure 3:**
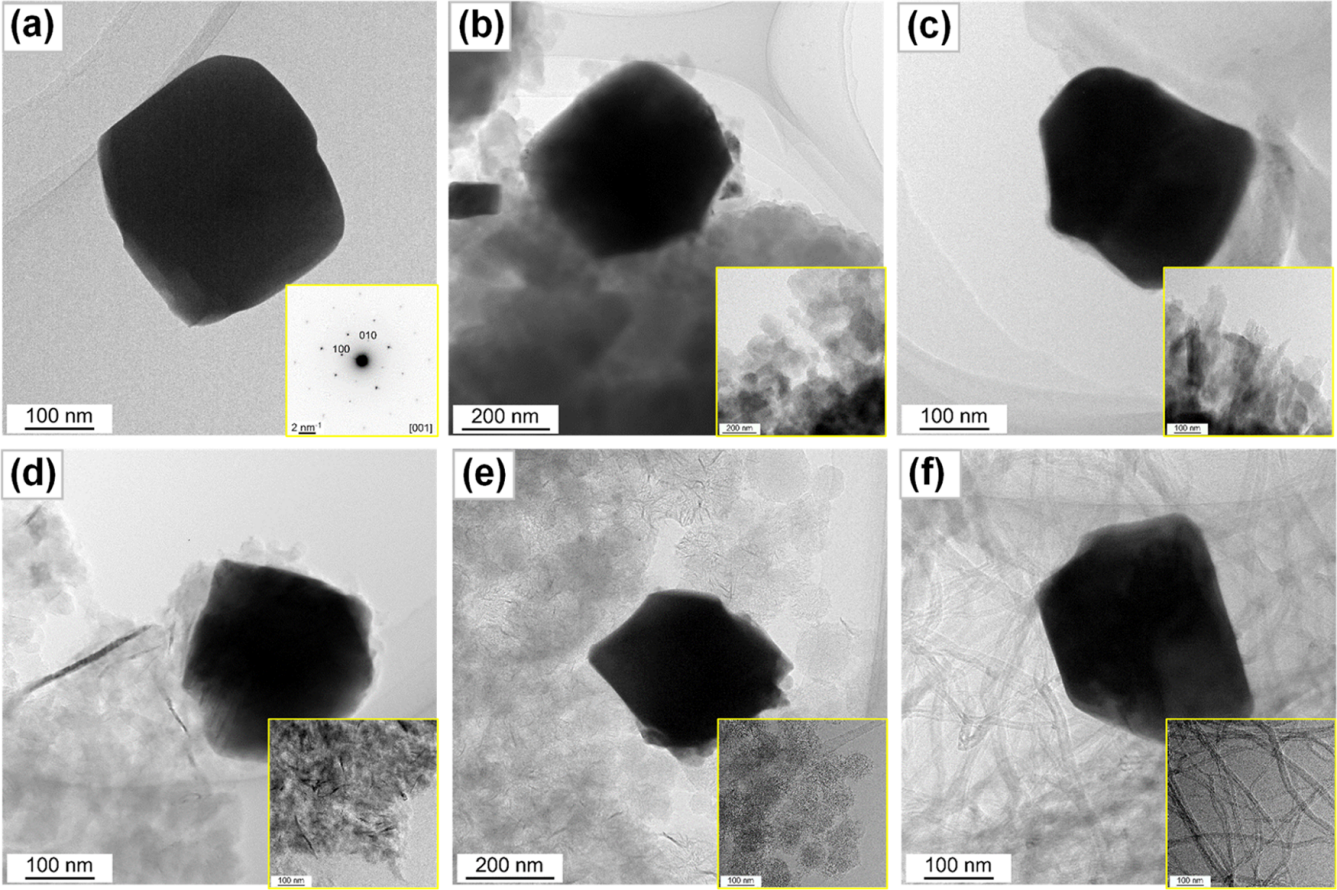
TEM images of BT (a), BT-FU (b), BT-CN (c), BT-GR (d), BT-NH (e), and BT-NT (f).

Afterward, the synthesized BaTaO_2_N powders were mechanically mixed with carbonaceous materials having different dimensions and examined by scanning and transmission electron microscopies. The SEM images in Figure 2 show that the synthesized BaTaO_2_N powders have an idiomorphic crystal morphology and an average crystal size of 286 nm. Apparently, some BaTaO_2_N crystals are aggregated, creating a high number of grain boundaries that may hinder majority carrier charge transport.^37^ On the contrary, Yabuta et al.^38^ pointed out that grain boundaries in aggregated particles do not act as recombination centers for photoexcited charge carriers but contribute to the prolongation of carrier lifetime. The BaTaO_2_N crystal is surrounded by facets. Particularly, the {110} planes exhibit edges or planes as facets as can be seen in Figure 3a. This morphological behavior suggests that BaTaO_2_N crystals have a high crystallinity. Despite their mechanical mixing, BaTaO_2_N crystals have close contact with the involved carbonaceous materials, such as fullerene, g-C_3_N_4_, graphene, carbon nanohorns, and carbon nanotubes (Figure 3). Such contacts are anticipated to facilitate the separation and transfer of photoexcited charge carriers in BaTaO_2_N crystals, promoting photoelectrochemical water oxidation performance.

**Figure 4:**
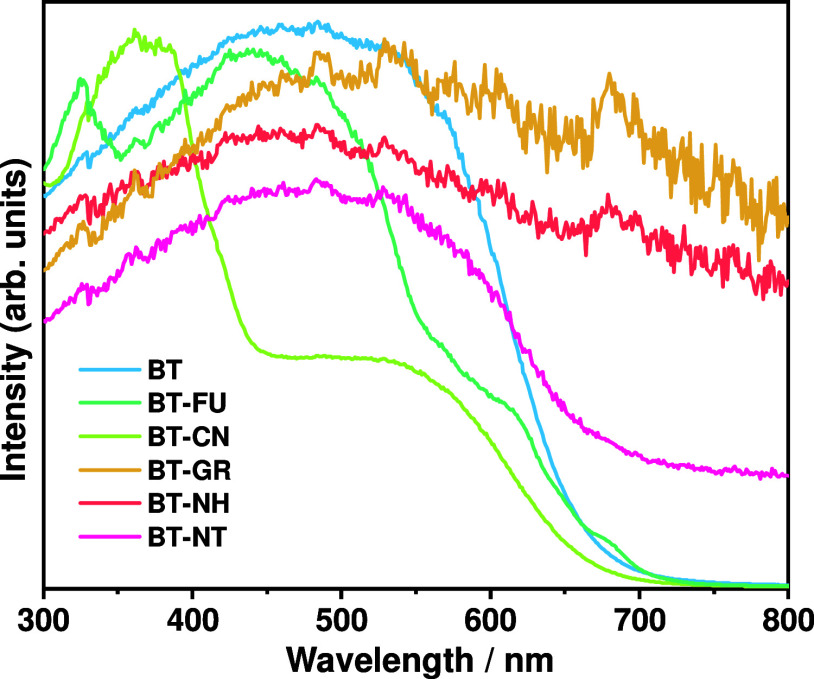
UV–vis diffuse reflectance spectra of BT, BT-FU, BT-CN, BT-GR, BT-NH, and BT-NT.

Figure 4 shows the UV–vis diffuse reflectance spectra of BT, BT-FU, BT-CN, BT-GR, BT-NH, and BT-NT samples. BaTaO_2_N powders (sample BT) indicate an onset of light absorption at a wavelength of approximately 665 nm, which corresponds to an optical band-gap energy of 1.87 eV. No background absorption beyond the absorption-edge wavelength is observed, suggesting the low defect density. Unlike the spectrum of the sample BT, the BT-FU and BT-CN samples exhibit two and three absorption edges in their corresponding UV–vis diffuse reflectance spectra, respectively. For BT-FU, three absorption edges located at the wavelengths of 700, 665, and 600 nm are associated with the light absorption edges of BaTaO_2_N powders and fullerene.^39^ For the BT-CN, two absorption edges positioned at the wavelengths of 665 and 460 nm are related to the light absorption edges of BaTaO_2_N powders and g-C_3_N_4_, respectively.^40^ The BT-GR, BT-NH, and BT-NT samples show light absorption with different intensities beyond the absorption-edge wavelength of BaTaO_2_N due to the response of graphene,^41^ carbon nanohorns,^42^ and carbon nanotubes^43^ to visible light beyond 665 nm. This implies that the BT-GR, BT-NH, and BT-NT samples have the capability to absorb more visible light beyond 665 nm in comparison to other samples.

### 3.2 Photoelectrochemical Performance

**Figure 5:**
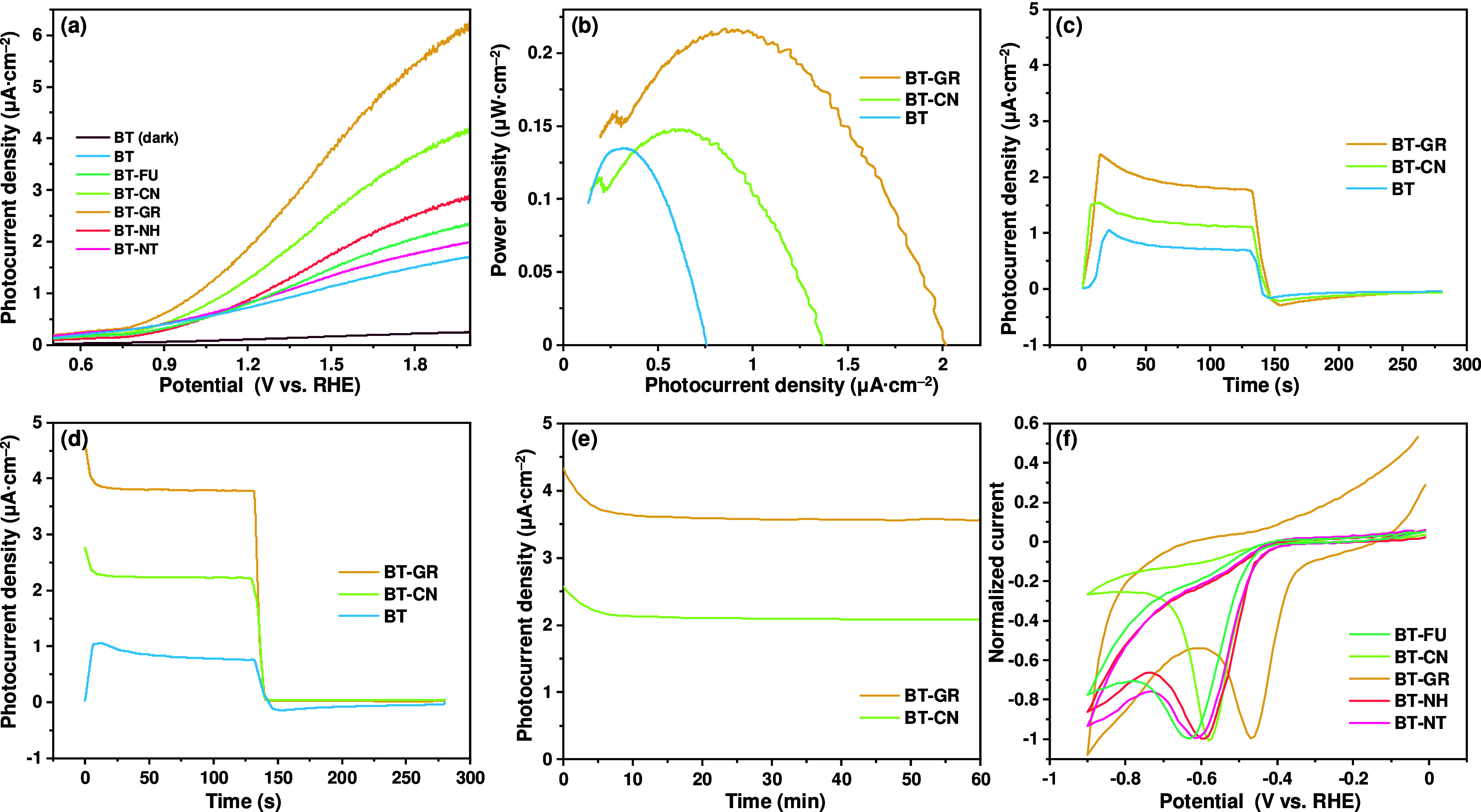
Photocurrent density vs potential (a), powder density vs photocurrent density (b), photocurrent density vs time under illumination–dark cycle at 1.2 V vs RHE (c) and 1.5 V vs RHE (d), photocurrent density vs time at 1.5 V vs RHE for 60 min (e), and normalized current vs potential (dark condition) (f).

Combining the BaTaO_2_N powders with carbonaceous materials with different dimensions in the fabrication of photoanodes is a straightforward strategy to understand the photoelectrochemical behavior of photocatalysts that hinder the collection and transfer of photoexcited charge carriers.^27,28^Figure 5a shows the LSV results of the BT, BT-FU, BT-CN, BT-GR, BT-NH, and BT-NT photoanodes. With the addition of each carbonaceous material, a relevant difference in the photocurrent densities was observed. Particularly, the difference in the photocurrent densities of BT-GR, BT-CN, and BT is obvious. Interestingly, no significant difference was observed for BT-FU, BT-NH, and BT-NT in the range of 0.6 and 1.2 V vs RHE. However, a trend in the photocurrent density in the range of 1.2 and 2.0 V vs RHE was elucidated. At high overpotentials, an increasing trend in the photocurrent densities was noted in the following order: 0.723 μA cm^–2^ (BT) < 0.802 μA cm^–2^ (BT-FU) < 0.816 μA cm^–2^ (BT-NT) < 0.874 μA cm^–2^ (BT-NH) < 1.279 μA cm^–2^ (BT-CN) < 1.879 μA cm^–2^ (BT-GR). Clearly, the BT-GR and BT-CN samples exhibited 2.6-fold and 1.7-fold higher photocurrent densities at 1.2 vs RHE compared to the BT sample. These results are consistent with previous reports on other types of photoanodes based on BiVO_4_/carbon spheres^27^ and WO_3_/nanoporous carbon.^28^ Gomis-Berenguer et al.^28,44^ observed a varying trend in the photoelectrochemical response of WO_3_/nanoporous carbon-based photoanodes for photocatalytic water oxidation with different weight ratios of nanoporous carbon, and 20 wt % was found to be the most favorable content of nanoporous carbon. It is noteworthy to mention that the enhanced PEC performance of BT-CN, beyond the intrinsic properties of carbonaceous material, is anticipated not solely due to its typical characteristics but also because of the synergistic photoelectrochemical response arising from the inherent photocatalytic processes exhibited by g-C_3_N_4_.^32,45,46^ Also, the photoactive nature of g-C_3_N_4_ contributes to the observed improvements, creating a distinctive charge accumulation region within the BT-CN composite.^45^ Consequently, the photocatalytic behavior of g-C_3_N_4_ significantly influences and augments the overall photoelectrochemical response in the BT-CN composite, further enhancing its performance. Furthermore, it has been reported that in both darkness and light,^46,47^ g-C_3_N_4_ can promote the oxygen reduction reaction (ORR), and the arguments to be presented later on ORR remain consistent for the BT-CN.

It should be noted that at low overpotentials, the photocurrent density of BaTaO_2_N (sample BT) is limited by the recombination and dynamics of photoexcited charge carriers.^11^ With the incorporation of graphene and g-C_3_N_4_, the photocurrent density of BaTaO_2_N at lower overpotentials was improved, and the onset potentials for BT-GR and BT-CN were 0.8 and 0.9 V vs RHE, respectively. Therefore, the improved photocurrent density provides evidence that carbonaceous materials can lower the recombination rate of photoexcited charge carriers. Gomis-Berenguer et al.^28^ enhanced the photoelectrochemical performance of hexagonal and monoclinic WO_3_ by incorporating them with nanoporous carbon, resulting in an increased photocurrent even at low overpotentials due to the lowered recombination rate stemming from the delocalization of electrons in nanoporous carbon. Also, the IPCEs of hexagonal and monoclinic WO_3_ were enhanced two and three times at different potentials and with different amounts of nanoporous carbon, respectively. In another study, Wang et al.^27^ employed carbon spheres as an electron reservoir, which was fed by electrons photoexcited in the BiVO_4_ photocatalyst, improving the photoelectrochemical performance substantially and leading to the photocurrent density of 2.20 mA cm^–2^ at 1.0 V vs RHE, which is about 6.5 times larger than the photocurrent density obtained for the BiVO_4_ photoanode. Considering that the BT-GR and BT-CN samples exhibit a notably higher photocurrent even at low overpotentials, a comparative discussion with BT is made based on chronoamperometric results and power density curves versus photocurrent density. Other photocatalysts do not show a large difference in the resulting photocurrent at 1.2 V vs RHE.

Figure 5b presents the power density vs photocurrent density curves for BT, BT-GR, and BT-CN photoanodes. In all cases, a parabolic-shaped contour is resolved, indicating the existence of a photocurrent density value where power density is maximized. The obtained results lead to finding the best possible operating conditions in the photoelectrochemical water-splitting cells. Since the photocurrent density is greater at high overpotentials, the application of electrochemical assistance can be an effective strategy to improve the photoelectrochemical performance of BaTaO_2_N. The external assistance of a photoelectrochemical cell at the photocurrent density that maximizes the power density suggests operating conditions where the transformation of solar energy into hydrogen is greater.^35^ Another relevant aspect of Figure 5b is that the difference between the behavior of the fabricated photoanodes is increased, giving clear evidence that graphene and g-C_3_N_4_ can outperform in enhancing the photoelectrochemical performance of BaTaO_2_N in comparison to other carbonaceous materials used in this study. In fact, the photocurrent density (power density) that maximized the solar-to-chemical energy conversion efficiency is 0.30 μA cm^–2^ (0.13 μW cm^–2^) for BT, which was increased to 0.91 μA cm^–2^ (0.22 μW cm^–2^) for BT-GR and 0.61 μA cm^–2^ (0.15 μW cm^–2^) for BT-CN. These conditions are the ones that must be imposed externally to implement these photocatalysts in reactors where the photocatalysis is electrochemically assisted so that the hydrogen evolution reaction (HER) can conveniently close the charge balance at an appropriate cathode.

Figure 5c,d shows the CA results of the BT, BT-GR, and BT-CN samples. In all cases, the photocurrent density as a function of time is higher under light irradiation than in the dark, defining a transient where the photocurrent spike is observed at the onset of light irradiation. It should be noted that when the light is turned off, the photocurrent density drops abruptly, registering a negative overshoot. The observed behavior in the CA results is characteristic of semiconductor-electrolyte interfaces, where the recombination of photoexcited charge carriers is important.^48^ A similar behavior was previously observed for BaTaO_2_N^5,11,23^ and TiN-modified LaTiO_2_N^49^ photoanodes. Therefore, the described qualitative evidence from the CA analysis confirms that the incorporation of graphene and g-C_3_N_4_ into BaTaO_2_N changes the delicate balance between the electron transfer and recombination of photoexcited charge carriers. This favors an efficient electron transfer and a photocurrent collection. Particularly, the photocurrent density at low overpotentials increases because the photoexcited electrons pass into the carbonaceous materials, separating the charges and influencing the recombination rate. At high overpotentials, a greater tendency to transfer electrons to carbonaceous materials is evident. The obtained results are consistent with the behavior of graphene^50^ and g-C_3_N_4_^32^ in photocatalysis. At this point, it is important to clarify that the recombination processes can manifest in various ways. One manifestation is evident in the overall changes in the net photocurrent: a higher photocurrent corresponds to a reduced recombination rate. Another aspect of recombination is reflected in the transient current profiles during the light–dark cycles. As described earlier, the appearance of spikes upon light illumination and a negative overshoot upon light extinction indicate recombination via surface and deep-level states. Notably, both BT-GR and BT-CN exhibit an increased photocurrent compared to that of BT, suggesting a reduction in recombination. However, the presence of peaks and negative overshoots in the transient response or CA results of BT-GR, BT-CN, and BT indicates that the recombination processes involving surface states persist.

To verify the effect of the potential on the complex dynamics of charge carriers and the stability of the electrodes giving a higher photocurrent (BT-GR and BT-CN), chronoamperometric tests were performed first at different potentials and then for a longer time. Figure 5c,5d shows the respective chronoamperometric responses obtained at 1.2 and 1.5 V (vs RHE) during a light–dark cycle. As mentioned, the shape of the current transient is associated with the balance between the electrons collected and those lost by recombination, affecting the kinetics of charge carriers. Higher bias increases the driving force to extract photoexcited electrons, thereby minimizing the current spikes and overshoots typically observed in the chronoamperometric response under light–dark cycles. In addition, Figure 5e shows the transient current at high polarization for over 60 min, enabling the observation of that current value. Although the photocurrent value is initially higher, it reaches a steady state, demonstrating the stability of the fabricated photoanodes over time scales longer than the conducted PEC measurements.

It is confirmed that the photoexcited electrons move from BaTaO_2_N to the carbonaceous material upon light irradiation, boosting the charge separation and favoring the accumulation of photoexcited holes in the valence band of BaTaO_2_N. Subsequently, the photoelectrochemical performance for water oxidation is enhanced. Apparently, the improvement in the photoelectrochemical performance of BaTaO_2_N depends on the contact between carbonaceous materials and BaTaO_2_N. Furthermore, the efficiency of carbonaceous materials may be related to their ability to accumulate, transfer, and donate photoexcited electrons due to their surface chemistry, dimensions, and electrocatalytic properties.

The discussion of the results turns to an interpretative analysis of the observed trends. In particular, the alignment between the oxygen reduction reaction (ORR) potential in the dark and the increased photocurrent density uncovers a rational connection between an apparently contrasting phenomenon. This observation becomes crucial when considering the observed improvement, which suggests an underlying accumulation phenomenon. If the performance of the composite photocatalyst is enhanced by charge accumulation in the carbonaceous phase after light excitation, then the same composite should be able to differentiate in the dark by performing reduction processes. To validate this concept, we turn to the compelling evidence provided by the ORR. Therefore, cyclic voltammetry (CV) was performed in the dark in the presence of the same electron acceptor (dissolved O_2_) for carbonaceous material-modified BaTaO_2_N photoanodes.

The CV results are listed in Figure 5f. The electrochemical response arises from the juxtaposition of two signals: (i) the capacitive signal from the fabricated photoanodes^51^ and (ii) the signal due to the oxygen reduction reaction (ORR).^52^ The ORR peak potential values were determined to be −0.46, −0.58, −0.60, −0.61, and −0.63 V vs RHE for BT-GR, BT-CN, BT-NH, BT-NT, and BT-FU, respectively. The obtained potential values are closer to the value of the reduction potential (one electron) of O_2_ (−0.33 V vs RHE^52^) and can be assigned to higher electrocatalytic activity for the above-mentioned reaction.^53^ Then, the BaTaO_2_N/carbonaceous materials are expected to be a better electron donor compared to BaTaO_2_N. Thus, when the photoanodes fabricated based on the combination of BaTaO_2_N and carbonaceous material are illuminated and subjected to an electrochemical gradient, it is anticipated that the extraction of electrons can be favored. For all photoanodes, the intensifying tendency in the photocurrent density is consistent with the trend measured for the ORR potential in the dark. That is, the more positive the ORR potential, the more likely the BaTaO_2_N/carbonaceous materials are to be better electron donors. Therefore, the reduction processes during photocatalysis must be improved. The findings from this study can be used as a descriptor to optimize the design of novel photocatalytic materials based on photocatalysts and carbonaceous materials.

Finally, it is worth noting that the increase in the photocurrent and the decrease in the onset potential observed for BT-GR and BT-CN are related to the respective good and large contact between BaTaO_2_N and graphene or g-C_3_N_4_. In addition to the fact that both graphene and g-C_3_N_4_ have high electron accumulation and charge retention properties, these characteristics are particularly associated with their two-dimensional structures.^54,55^ Therefore, the depolarization of these carbonaceous materials, which are enriched with electrons during the photocatalytic process, depends on their electrocatalytic activity. It can be stated that the electrocatalytic performance of graphene and g-C_3_N_4_ is relatively high in both ORR and water reduction reactions.^56,57^ The fact that BT-GR defines a higher photocurrent density than BT-CN, at both high and low overpotentials, may also be related to the electrochemical properties of graphene^58,59^ and g-C_3_N_4_.^60,61^ It is inferred that the higher conductivity and electron transfer properties of graphene^58,59^ in comparison to that of g-C_3_N_4_^60^ increase the efficiency of electron collection during the photoelectrochemical reaction in the presence of BaTaO_2_N (Figure 5a,5c,d). Thus, graphene assists in lowering the energy loss in BT-GR. In all cases, the observed transduction of the properties of graphene and g-C_3_N_4_ to BT-GR and BT-CN photoanodes could enhance the photoelectrochemical performance of BaTaO_2_N.

**Figure 6:**
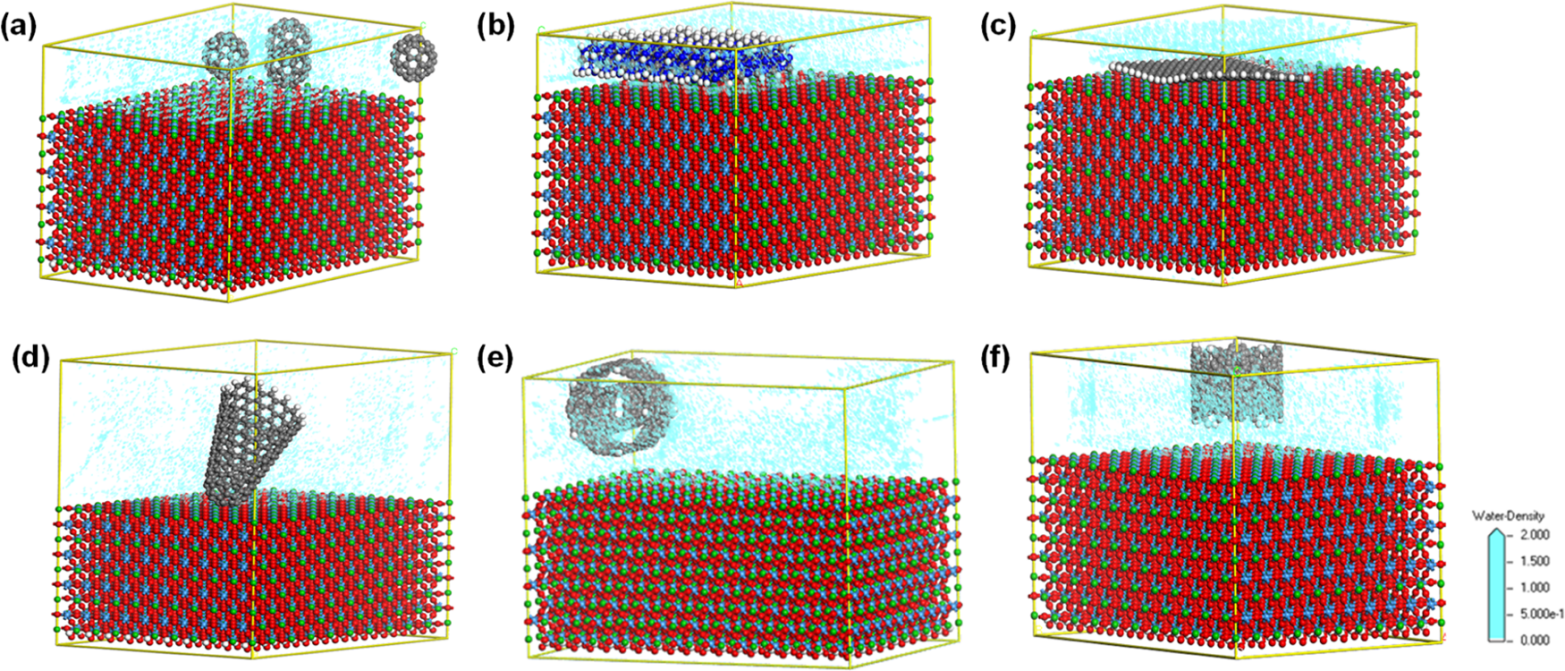
Visualization of the adsorption of water molecules onto BT-FU (a), BT-CN (b), BT-GR (c), BT-NH (d), BT-NT-horizontal (e), and BT-NT-vertical (f). The green, light blue, red, dark blue, and gray colors represent barium, tantalum, oxygen, nitrogen, and carbon, respectively. Isosurface field density: water in turquoise blue.

**Figure 7:**
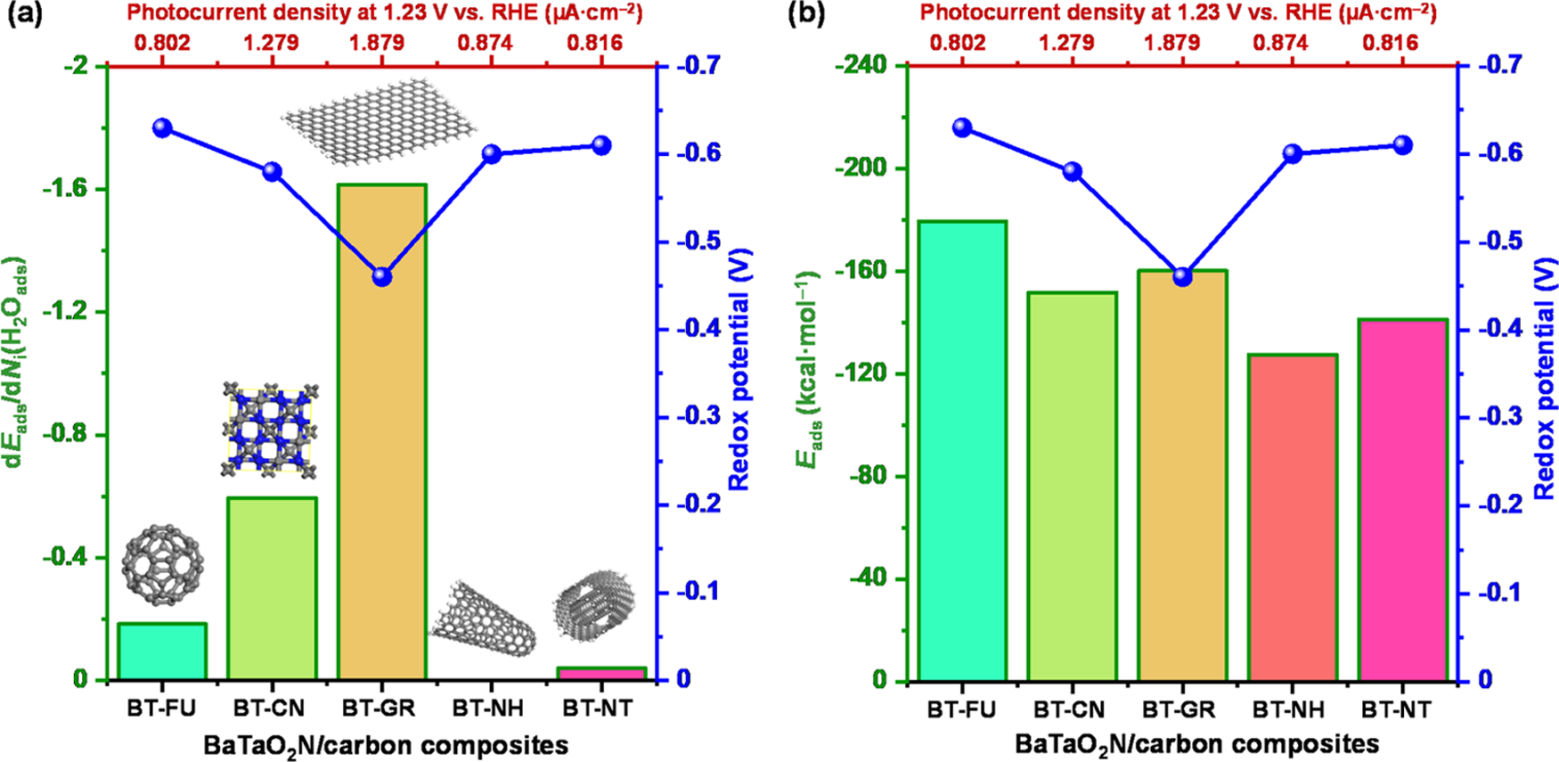
(a) Relationship between differential energy of adsorption of water molecules, redox potential, and photocurrent density and (b) relationship between the energy of adsorption of water molecules, redox potential, and photocurrent density of BT-FU, BT-CN, BT-GR, BT-NH, and BT-NT composites.

**Figure 8:**
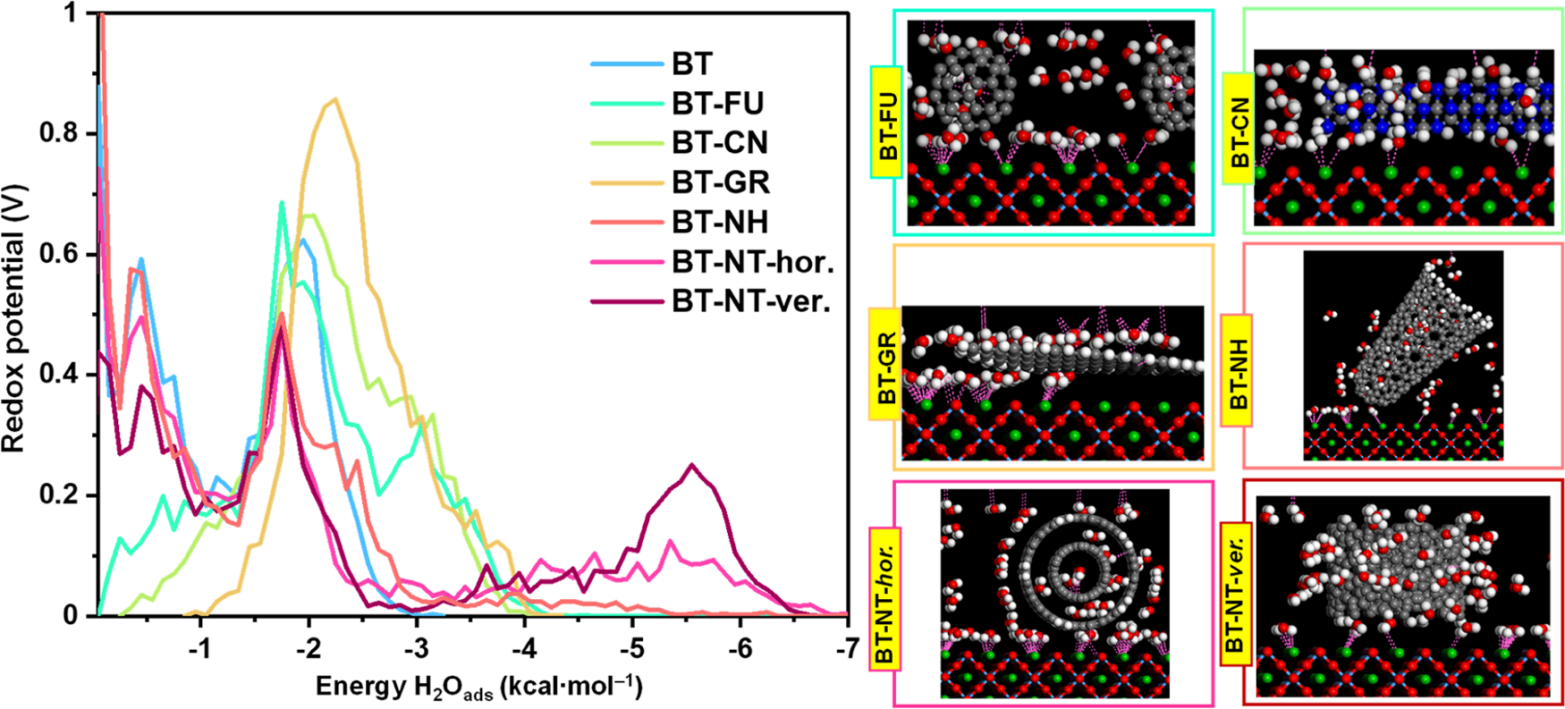
(a) Energy distribution of adsorbed water molecules and (b) formation of water molecule layers on BT-FU, BT-CN, BT-GR, BT-NH, and BT-NT composites. The green, light blue, red, dark blue, and gray colors represent barium, tantalum, oxygen, nitrogen, and carbon, respectively.

The surface chemistry and interfacial effects between heterogeneous photocatalysts and surface water molecules are important for efficient solar water splitting. The electronic and structural properties of photocatalyst/water and photocatalyst/carbonaceous material/water interfaces depend on the electronic structure of a photocatalyst and band potentials matching with water redox potentials.^62^ The specific aqueous interface structure, chemical surface complexation, and the structure and morphology of nanocrystals have significant impacts on the band-edge positions of photoelectrode materials, Ohmic contacts, and Schottky barriers. In addition, the hydrophilic surfaces with surface oxygen vacancies at the terminated surfaces of metal oxides can form a hydrated layer on the surfaces and interfaces of photoelectrodes. However, it should be mentioned that it is still difficult to investigate the adsorption of water molecules using heterogeneous, multiatomic structure interface models by high-cost, first-principles molecular dynamics (FPMD) and density functional theory (DFT) simulations. Using implicit-solvent models with the Monte Carlo method provides a faster and inexpensive approach to exploring the interaction of the photocatalyst surface/interface with water molecules with regard to the surface photocatalytic reaction, which is strongly dependent on the stability of the photocatalyst, the adsorption of water molecules, and the formation of clusters on the photocatalyst surface.^63^ Therefore, the structure and adsorption energetics of the BaTaO_2_N/carbonaceous material interface and the interaction with water molecules were studied using the Monte Carlo method (BIOVIA, Adsorption Locator module)^64,65^ and experimental structural data,^36,66^ and the results are presented in Figures 6, 7, and 8.

The modeled composites consisted of BaTaO_2_N (BT) with a crystallographic plane of (110) and different carbonaceous materials: g-C_3_N_4_ (CN) with a crystallographic plane of (002), multiwalled carbon nanotubes (NT), C60-fullerene (FU), carbon nanohorns (NHs), or graphene (GR) in the same periodic box. The predominant crystallographic planes of BaTaO_2_N and C_3_N_4_ were confirmed by the XRD results. The layer of carbonaceous materials consisted of 250 carbon atoms, except for 240 carbon atoms for fullerene. The direct contact of carbonaceous materials and BaTaO_2_N is strongly related to the morphology of carbonaceous materials. The decrease in the values of the carbon surface affinity (*E*_BT/C_) in the composite indicates the stability of the BT/carbon interface (Table 1). The BT-GR composite has the most stable interface interaction (−221.29 kcal mol^–1^) compared with BT-FU (−97.75 kcal mol^–1^), BT-CN (−156.71 kcal mol^–1^), BT-NH (−58.75 kcal mol^–1^), and BT-NT (−40.25 kcal mol^–1^). The affinities of water molecules were evaluated by distribution field density (Figure 6), differential energy of adsorption of water molecules (Figure 7), and energy distribution of water molecules (Figure 8) adsorbed between the predominant surface of BaTaO_2_N (110) and carbonaceous materials with different morphologies: spherical (buckyballs) C_60_-fullerene, single-sheet graphene, two-dimensional (2D) layered graphite-like structure (g-C_3_N_4_ (002)), tubular multiwalled carbon nanotube, and conical single-walled carbon nanohorn.

**Table 1:** Energy Parameters of the Adsorption of Water Molecules

structure	water adsorption energy, *E*_ads_ (kcal mol^–1^)	d*E*_ads_/d*N_i_*	carbon surface affinity, *E*_BT/C_ (kcal mol^–1^)
BaTaO_2_N (110)	–98.42	–0.01	
BT-FU	–179.56	–0.18	–97.75
BT-CN	–151.76	–0.59	–156.71
BT-GR (002)	–160.20	–1.61	–221.28
BT-NH	–127.55	–0.01	–58.75
BT-NT (horizontal)	–202.52	–0.01	–40.25
BT-NT (vertical)	–245.58	–0.06	

Figure 6 shows the interfaces between BaTaO_2_N and carbonaceous materials and the formation of a stable layer of adsorbed molecules on the composite surface. The distribution field density of adsorbed water molecules depends on the localization of the carbon nanoparticles. Carbon nanoparticles have a random spreading in the periodic box except for carbon nanotubes modeled in horizontal (Figure 6e) and vertical (Figure 6f) positions. Generally, the addition of carbonaceous materials increased the *E*_ads_ value of BaTaO_2_N (−98.42 kcal mol^–1^) (Table 1 and Figure 7b), and the highest *E*_ads_ values were observed for the BT-NT composite with carbon nanotubes placed in horizontal and vertical positions to the surface of BaTaO_2_N. The calculated *E*_ads_ values have the following order: −245.58 kcal mol^–1^ for BT-NT (vertical) < −202.52 kcal mol^–1^ for BT-NT (horizontal) < −179.57 kcal mol^–1^ for BT-FU < −160.21 kcal mol^–1^ for BT-GR < −151.76 kcal mol^–1^ for BT-CN < −127.56 kcal mol^–1^ for BT-NH < −98.43 kcal mol^–1^ for BT, while the differential adsorption has a different order: −1.61 kcal mol^–1^ for BT-GR < −0.60 kcal mol^–1^ for BT-CN < −0.18 kcal mol^–1^ for BT-FU < −0.06 kcal mol^–1^ for BT-NT (vertical) < −0.02 kcal mol^–1^ for BT-NT (horizontal) < −0.01 kcal mol^–1^ for BT = −0.01 kcal mol^–1^ for BT-NH. The highest value of differential adsorption (d*E*_ads_/d*N_i_*) is in good agreement with the experimental data (Figure 7a). Computational simulation and experimental data revealed that the water molecules can form stable hydrated multilayers on the flat units of graphene.^67^ The highest peak observed at an adsorption energy of −2.15 kcal mol^–1^ (Figure 8) indicates a large number of water molecules adsorbed on the surface of the BT-GR composite. In the cases of BT-FU, BT-NH, and BT-NT composites, the presence of multiple peaks noted at different adsorption energies confirms a number of water molecules adsorbed on different adsorption sites in comparison to the BT-GR and BT-CN composites.

However, the highest values of *E*_ads_ for BT-NT, BT-NH, and BT-FU (Figure 7b) are the subject of controversy regarding the mode of adsorption of water molecules. The increased value of *E*_ads_ can be explained by the impact of possible adsorption of a part of water molecules in the internal channel of nanotubes, nanohorns, and fullerene.^67−70^ It is known that the carbon nanotube channel is strongly hydrophobic but can filled up with water molecules forming a hydrogen-bonded chain.^69^ The structure of carbon nanohorns has a closed horn tip at one side and an open end on the other side, allowing the water molecules to enter.^69^ Although fullerene is hydrophobic, the C60 nanoclusters have a hydrophilic nature that can increase the *E*_ads_ value of BT-FU.^70^ The water molecules prefer to be adsorbed around the intrinsic vacancies of a single sheet of g-C_3_N_4_ as clusters. The adsorption of water molecules on both sides of g-C_3_N_4_ does not affect the flat structure. However, the adsorption of water molecules on one side of a single g-C_3_N_4_ sheet leads to the transformation of the flat structure with indirect semiconductor properties to buckle the structure with direct semiconductor properties. Therefore, the band structure of g-C_3_N_4_ is finally changed due to the adsorption of water molecules.^7^ A previous study^62^ found that the exothermic adsorption of water molecules in the presence of chemisorbed oxygen causes the dissociation of water molecules on the hydroxylated surfaces of photocatalysts via a partial proton transfer mechanism at the interface. In addition, the surface anions can accept protons from water molecules, while equivalent OH ions form bonds with surface cations. The N-sites can also be protonated even more rapidly than the surface oxygen sites.^62^ Finally, the water molecules can be adsorbed dissociatively on the surface oxygen vacancies, leading to the formation of surface hydroxyls and oxidative intermediates (H* for H_2_ evolution and HO*, O*, and HOO* for O_2_ evolution).^7^

The dissociation of water molecules is dependent on the termination of the photocatalyst surface. BaTaO_2_N has negative charges on the oxygen-terminated surfaces and positive charges on the barium-terminated surfaces. The water molecules prefer to be adsorbed by interaction with surface oxygen and on the top side of the Ba atom on the (110) surface, forming the Ta–O···H_2_O and H_2_O···Ba bonds because the coordination of water molecules to the BaTaO_2_N surface is energetically more favorable than to carbonaceous materials due to electrostatic forces. The adsorption of water oxygen atoms located on the exposed cation and anion sites varies less proportionally to the number of oxygen atoms missing from the normal Ta(O,N_6_) octahedral coordination and the Ba cation, which can be 12-fold surrounded by anions. The most stable surfaces are shown to be along the (110) crystallographic planes that have more metal cations exposed. The analysis of energy distribution of adsorbed water molecules (Figure 8) confirmed the shift to higher adsorption energy in the case of the BT-GR composite due to the interaction of the conjugated π-system of graphene and the BaTaO_2_N surface. Although the BT-NT (horizontal), BT-NH, and BT-FU composites also have the possibility to form similar interactions, the BT-GR composite has a more pronounced effect on the ability of water adsorption.

The adsorption of water molecules on the BaTaO_2_N/carbonaceous material surfaces was found to be a favorable exothermic process. The surface reactivity of the BT-GR composite is significantly higher in comparison to that of BT-FU, BT-NT, and BT-NH, and the possible dissociation of water molecules is highly favorable on the BT-GR and BT-CN surfaces. The experimental results confirmed that the ORR potential is correlated with the photocurrent density and adsorption of water molecules at the interface between BaTaO_2_N and carbonaceous materials (Figure 7). Considering the localization of the distribution field density of adsorbed water molecules, it is clear that the adsorption of water molecules is favored onto surfaces of BT-GR and BT-CN composites. Thus, the incorporation of graphene can lead to a more positive ORR potential and better electron donors, which should be in line with the photoredox flux-matching conditions during the photocatalysis process, suggesting the improvement of the reduction processes during photocatalysis.^71^ Therefore, the BT-GR composite exhibited a higher water oxidation reaction (WOR) performance.

## 4 Conclusions

In this study, the photoelectrochemical performance of BaTaO_2_N was enhanced by involving carbonaceous materials, such as fullerene, g-C_3_N_4_, graphene, carbon nanohorns, and carbon nanotubes. The difference in the photoelectrochemical performance of BaTaO_2_N/carbonaceous materials and BaTaO_2_N could be attributed to the capability of each carbonaceous material for the collection and transfer of photoexcited electrons, thus decreasing the recombination of photoexcited charge carriers. Particularly, the photocurrent density of BaTaO_2_N was 2.6- and 1.7-fold increased using graphene and g-C_3_N_4_ due to the efficient electron transfer, electron reservoir capacity, and accumulation of a greater number of photoexcited holes on the valence band. The comparison of trends between the ORR potential in the dark and the increase in the photocurrent density validates the charge accumulation process in the carbonaceous phase, highlighting that the subsequent photocurrent collection was not compromised. This implies that the more positive the ORR potential is, the better electron donors the BaTaO_2_N/carbonaceous materials are expected to be, suggesting the improvement of the reduction processes during photocatalysis. Computational simulation of the adsorption of water molecules onto the surfaces of BaTaO_2_N/carbonaceous materials revealed that the incorporation of graphene can enhance the water oxidation reaction performance of BaTaO_2_N. The photoelectrochemical performance of other photocatalysts can also be improved using carbonaceous materials having different dimensions, morphologies, and surface and optoelectronic properties.

## References

[ref1] Nozik A. J. Photoelectrochemistry: Applications to Solar Energy Conversion. Annu. Rev. Phys. Chem. 1978, 29, 189–222. 10.1146/annurev.pc.29.100178.001201.

[ref2] Grätzel M. Photoelectrochemical cells. Nature 2001, 414, 338–344. 10.1038/35104607. 11713540

[ref3] Klahr B.; Gimenez S.; Fabregat-Santiago F.; Bisquert J.; Hamann T. W. Electrochemical and Photoelectrochemical Investigation of Water Oxidation with Hematite Electrodes. Energy Environ. Sci. 2012, 5, 7626–7636. 10.1039/c2ee21414h.

[ref4] Higashi M.; Domen K.; Abe R. Fabrication of an Efficient BaTaO_2_N Photoanode Harvesting a Wide Range of Visible Light for Water Splitting. J. Am. Chem. Soc. 2013, 135, 10238–10241. 10.1021/ja404030x. 23808352

[ref405] Hojamberdiev M.; Vargas R.; Zhang F.; Teshima K.; Lerch M. Perovskite BaTaO_2_N: From Materials Synthesis to Solar Water Splitting. Adv. Sci. 2023, 10, 2305179 10.1002/advs.202305179. PMC10667847 37852947

[ref5] Ueda K.; Minegishi T.; Clune J.; Nakabayashi M.; Hisatomi T.; Nishiyama H.; Katayama M.; Shibata N.; Kubota J.; Yamada T.; Domen K. Photoelectrochemical Oxidation of Water Using BaTaO_2_N Photoanodes Prepared by Particle Transfer Method. J. Am. Chem. Soc. 2015, 137, 2227–2230. 10.1021/ja5131879. 25650748

[ref6] Chen Z.; Dinh H. N.; Miller E. Photoelectrochemical Water Splitting: Standards, Experimental Methods, and Protocols; Springer: New York, 2013.

[ref7] Hojamberdiev M.; Vargas R.; Kadirova Z. C.; Sena H.; Krasnov A.; Teshima K.; Lerch M.; et al. Unfolding the Role of B-Site-Selective Doping of Aliovalent Cations on Enhancing Sacrificial Visible-Light-Induced Photocatalytic H_2_ and O_2_ Evolution over BaTaO_2_N. ACS Catal. 2022, 12, 1403–1414. 10.1021/acscatal.1c04547.

[ref8] Hojamberdiev M.; Vargas R.; Kadirova Z. C.; Teshima K.; Lerch M. Exploring the Effect of B-Site Al^3+^-Mg^2+^ Dual Substitution on Optoelectronic, Surface, and Photocatalytic Properties of BaTaO_2_N. Mater. Adv. 2022, 3, 7348–7359. 10.1039/D2MA00611A.

[ref9] Hojamberdiev M.; Zahedi E.; Nurlaela E.; Kawashima K.; Yubuta K.; Nakayama M.; Wagata H.; Minegishi T.; Domen K.; Teshima K. The Cross-Substitution Effect of Tantalum on Visible-Light-Driven Water Oxidation Activity of BaNbO_2_N Crystals Grown Directly by an NH_3_-Assisted Flux Method. J. Mater. Chem. A 2016, 4, 12807–12817. 10.1039/C6TA03786K.

[ref10] Zhang Y.; Xu X. LaTaON_2_–BaTaO_2_N solid solutions for photocatalytic water oxidation. Inorg. Chem. Front. 2021, 8, 3723–3732. 10.1039/D1QI00598G.

[ref11] Hojamberdiev M.; Mora-Hernandez J. M.; Vargas R.; Yamakata A.; Yubuta K.; Heppke E. M.; Torres-Martínez L. M.; Teshima K.; Lerch M. Time-retrenched synthesis of BaTaO_2_N by localizing an NH_3_ delivery system for visible-light-driven photoelectrochemical water oxidation at neutral pH: Solid-state reaction or flux method?. ACS Appl. Energy Mater. 2021, 4, 9315–9327. 10.1021/acsaem.1c01539.

[ref12] Li H.; Xiao J.; Vequizo J. J. M.; Hisatomi T.; Nakabayashi M.; Pan Z.; Shibata N.; Yamakata A.; Takata T.; Domen K. One-Step Excitation Overall Water Splitting over a Modified Mg-Doped BaTaO_2_N Photocatalyst. ACS Catal. 2022, 12, 10179–10185. 10.1021/acscatal.2c02394.

[ref13] Hojamberdiev M.; Yubuta K.; Vequizo J. J. M.; Yamakata A.; Oishi S.; Domen K.; Teshima K. NH_3_-Assisted Flux Growth of Cube-Like BaTaO_2_N Submicron Crystals in a Completely Ionized Nonaqueous High-Temperature Solution and Their Water Splitting Activity. Cryst. Growth Des. 2015, 15, 4663–4671. 10.1021/acs.cgd.5b00927.

[ref14] Teshima K.; Hara Y.; Yubuta K.; Oishi S.; Domen K.; Hojamberdiev M. Application of Flux Method to the Fabrication of Ba_5_Ta_4_O_15_, Sr_5_Ta_4_O_15_, Sr_2_Ta_2_O_7_, and BaTaO_2_N Polycrystalline Films on Ta Substrates. Cryst. Growth Des. 2017, 17, 1583–1588. 10.1021/acs.cgd.6b01573.

[ref15] Haydous F.; Döbeli M.; Si W.; Waag F.; Li F.; Pomjakushina E.; Wokaun A.; Gökce B.; Pergolesi D.; Lippert T. Oxynitride Thin Films versus Particle-Based Photoanodes: A Comparative Study for Photoelectrochemical Solar Water Splitting. ACS Appl. Energy Mater. 2019, 2, 754–763. 10.1021/acsaem.8b01811.

[ref16] Hojamberdiev M.; Kawashima K.; Hisatomi T.; Katayama M.; Hasegawa M.; Domen K.; Teshima K. Distinguishing the effects of altered morphology and size on visible-light-induced water oxidation activity and photoelectrochemical performance of BaTaO_2_N crystal structures. Faraday Discus. 2019, 215, 227–241. 10.1039/C8FD00170G. 30984922

[ref17] Luo Y.; Wang Z.; Yamada T.; Yubuta K.; Suzuki S.; Hisatomi T.; Domen K.; Teshima K. Platy BaTaO_2_N Crystals Fabricated from K_2_CO_3_–KCl Binary Flux for Photocatalytic H_2_ Evolution. ACS Appl. Energy Mater. 2020, 3, 10669–10675. 10.1021/acsaem.0c01739.

[ref18] Luo Y.; Suzuki S.; Wang Z.; Yubuta K.; Vequizo J. J. M.; Yamakata A.; Shiiba H.; Hisatomi T.; Domen K.; Teshima K. Construction of Spatial Charge Separation Facets on BaTaO_2_N Crystals by Flux Growth Approach for Visible-Light-Driven H_2_ Production. ACS Appl. Mater. Interfaces 2019, 11, 22264–22271. 10.1021/acsami.9b03747. 31150579

[ref19] Maeda K.; Domen K. Water Oxidation Using a Particulate BaZrO_3_-BaTaO_2_N Solid-Solution Photocatalyst That Operates under a Wide Range of Visible Light. Angew. Chem., Int. Ed. 2012, 51, 9865–9869. 10.1002/anie.201204635. 22951897

[ref20] Higashi M.; Yamanaka Y.; Tomita O.; Abe R. Fabrication of Cation-Doped BaTaO_2_N Photoanodes for Efficient Photoelectrochemical Water Splitting Under Visible Light Irradiation. APL Mater. 2015, 3, 104418 10.1063/1.4931487.

[ref21] Wei S.; Chang S.; Yang F.; Fu Z.; Liu G.; Xu X. Stable and efficient solar-driven photoelectrochemical water splitting into H_2_ and O_2_ based on a BaTaO_2_N photoanode decorated with CoO microflowers. Chem. Commun. 2021, 57, 4412–4415. 10.1039/D0CC07778J. 33949405

[ref22] Pihosh Y.; Nandal V.; Minegishi T.; Katayama M.; Yamada T.; Seki K.; Sugiyama M.; Domen K. Development of a Core-Shell Heterojunction Ta_3_N_5_-Nanorods/BaTaO_2_N Photoanode for Solar Water Splitting. ACS Energy Lett. 2020, 5, 2492–2497. 10.1021/acsenergylett.0c00900.

[ref23] Seo J.; Nakabayashi M.; Hisatomi T.; Shibata N.; Minegishi T.; Domen K. Solar-Driven Water Splitting over a BaTaO_2_N Photoanode Enhanced by Annealing in Argon. ACS Appl. Energy Mater. 2019, 2, 5777–5784. 10.1021/acsaem.9b00908.

[ref24] Liang Z.; Hou H.; Fang Z.; Gao F.; Wang L.; Chen D.; Yang W. Hydrogenated TiO_2_ Nanorod Arrays Decorated with Carbon Quantum Dots toward Efficient Photoelectrochemical Water Splitting. ACS Appl. Mater. Interfaces 2019, 11, 19167–19175. 10.1021/acsami.9b04059. 31058485

[ref25] Huang Y.; Liang Y.; Rao Y.; Zhu D.; Cao J.-j.; Shen Z.; Ho W.; Lee S. C. Environment-Friendly Carbon Quantum Dots/ZnFe_2_O_4_ Photocatalysts: Characterization, Biocompatibility, and Mechanisms for NO Removal. Environ. Sci. Technol. 2017, 51, 2924–2933. 10.1021/acs.est.6b04460. 28145696

[ref26] Xie X.; Yang Y.; Xiao Y.-H.; Huang X.; Shi Q.; Zhang W.-D. Enhancement of photoelectrochemical activity of Fe_2_O_3_ nanowires decorated with carbon quantum dots. Int. J. Hydrogen Energy 2018, 43, 6954–6962. 10.1016/j.ijhydene.2018.02.099.

[ref27] Wang M.; Wang Z.; Zhang B.; Jiang W.; Bao X.; Cheng H.; Zheng Z.; Wang P.; Liu Y.; Whangbo M.-H.; Li Y.; Dai Y.; Huang B. Enhancing the Photoelectrochemical Water Oxidation Reaction of BiVO_4_ Photoanode by Employing Carbon Spheres as Electron Reservoirs. ACS Catal. 2020, 10, 13031–13039. 10.1021/acscatal.0c03671.

[ref28] Gomis-Berenguer A.; Iniesta J.; Fermín D. J.; Ania C. O. Photoelectrochemical Response of WO_3_/Nanoporous Carbon Anodes for Photocatalytic Water Oxidation. C: J. Carbon Res. 2018, 4, 45 10.3390/c4030045.

[ref29] Zhao Y.; Zhang Y.; Wang Y.; Cao D.; Sun X.; Zhu H. Versatile zero- to three-dimensional carbon for electrochemical energy storage. Carbon Energy 2021, 3, 895–915. 10.1002/cey2.137.

[ref30] Kawashima K.; Hojamberdiev M.; Wagata H.; Yubuta K.; Oishi S.; Teshima K. Chloride Flux Growth of La_2_TiO_5_ Crystals and Nontopotactic Solid-State Transformation to LaTiO_2_N Crystals by Nitridation Using NH_3_. Cryst. Growth Des. 2015, 15, 333–339. 10.1021/cg501397x.

[ref31] Hojamberdiev M.; Khan M. M.; Kadirova Z.; Kawashima K.; Yubuta K.; Teshima K.; Riedel R.; Hasegawa M. Synergistic effect of g-C_3_N_4_, Ni(OH)_2_ and halloysite in nanocomposite photocatalyst on efficient photocatalytic hydrogen generation. Renewable Energy 2019, 138, 434–444. 10.1016/j.renene.2019.01.103.

[ref32] Velázquez L. S. G.; Dell’Arciprete M. L.; Madriz L.; Gonzalez M. C. Carbon nitride from urea: Mechanistic study on photocatalytic hydrogen peroxide production for methyl orange removal. Catal. Comm. 2023, 175, 106617 10.1016/j.catcom.2023.106617.

[ref33] Hojamberdiev M.; Czech B.; Wasilewska A.; Boguszewska-Czubara A.; Yubuta K.; Wagata H.; Daminova S. S.; Kadirova Z. C.; Vargas R. Detoxifying SARS-CoV-2 antiviral drugs from model real wastewaters by industrial waste-derived multiphase photocatalysis. J. Hazard. Mater. 2022, 429, 128300 10.1016/j.jhazmat.2022.128300. 35077970 PMC8767938

[ref34] Wang C.; Hisatomi T.; Minegishi T.; Wang Q.; Zhong M.; Katayama M.; Kubota J.; Domen K. Synthesis of nanostructured BaTaO_2_N thin films as photoanodes for solar water splitting. J. Phys. Chem. C 2016, 120, 15758–15764. 10.1021/acs.jpcc.5b11564.

[ref35] Madriz L.; Tatá J.; Carvajal D.; Núñez O.; Scharifker B. R.; Mostany J.; Borrás C.; Cabrerizo F. M.; Vargas R. Photocatalysis and photoelectrochemical glucose oxidation on Bi_2_WO_6_: Conditions for the concomitant H_2_ production. Renewable Energy 2020, 152, 974–983. 10.1016/j.renene.2020.01.071.

[ref36] Pors F.; Marchand R.; Laurent Y.; Bacher P.; Roult G. Etude structurale des perovskites oxyazotes BaTaO_2_N et BaNbO_2_N. Mater. Res. Bull. 1988, 23, 1447–1450. 10.1016/0025-5408(88)90270-X.

[ref37] Warren S. C.; Voïtchovsky K.; Dotan H.; Leroy C. M.; Cornuz M.; Stellacci F.; Hébert C.; Rothschild A.; Grätzel M. Identifying champion nanostructures for solar water-splitting. Nat. Mater. 2013, 12, 842–849. 10.1038/nmat3684. 23832125

[ref38] Yabuta M.; Takeda A.; Sugimoto T.; Watanabe K.; Kudo A.; Matsumoto Y. Particle Size Dependence of Carrier Dynamics and Reactivity of Photocatalyst BiVO_4_ Probed with Single-Particle Transient Absorption Microscopy. J. Phys. Chem. C 2017, 121, 22060–22066. 10.1021/acs.jpcc.7b06230.

[ref39] Peng B. Monolayer Fullerene Networks as Photocatalysts for Overall Water Splitting. J. Am. Chem. Soc. 2022, 144, 19921–19931. 10.1021/jacs.2c08054. 36260929 PMC9634807

[ref40] Ong W.-J.; Tan L.-L.; Ng Y. H.; Yong S.-T.; Chai S.-P. Graphitic Carbon Nitride (g-C_3_N_4_)-Based Photocatalysts for Artificial Photosynthesis and Environmental Remediation: Are We a Step Closer To Achieving Sustainability?. Chem. Rev. 2016, 116, 7159–7329. 10.1021/acs.chemrev.6b00075. 27199146

[ref41] Xie G.; Zhang K.; Guo B.; Liu Q.; Fang L.; Gon J. R. Graphene-Based Materials for Hydrogen Generation from Light-Driven Water Splitting. Adv. Mater. 2013, 25, 3820–3839. 10.1002/adma.201301207. 23813606

[ref42] Kagkoura A.; Tagmatarchis N. Carbon Nanohorn-Based Electrocatalysts for Energy Conversion. Nanomaterials 2020, 10, 1407 10.3390/nano10071407. 32707696 PMC7408240

[ref43] Ahmed A. M.; Mohamed F.; Ashraf A. M.; Shaban M.; Khan A. A. P.; Asiri A. M. Enhanced photoelectrochemical water splitting activity of carbon nanotubes@TiO_2_ nanoribbons in different electrolytes. Chemosphere 2020, 238, 124554 10.1016/j.chemosphere.2019.124554. 31421463

[ref44] Gomis-Berenguer A.; Celorrio V.; Iniesta J.; Fermin D. J.; Ania C. O. Nanoporous carbon/WO_3_ anodes for an enhanced water photooxidation. Carbon 2016, 108, 471–479. 10.1016/j.carbon.2016.07.045.

[ref45] Wang L.; Si W.; Tong Y.; Hou F.; Pergolesi D.; Hou J.; Lippert T.; Dou S. X.; Liang J. Graphitic carbon nitride (g-C_3_N_4_)-based nanosized heteroarrays: Promising materials for photoelectrochemical water splitting. Carbon Energy 2020, 2, 223–250. 10.1002/cey2.48.

[ref46] Gómez-Velázquez L. S.; Madriz L.; Rigoletto M.; Laurenti E.; Bizarro M.; Dell’Arciprete M. L.; González M. C. Structural and Physicochemical Properties of Carbon Nitride Nanoparticles via Precursor Thermal Treatment: Effect on Methyl Orange Photocatalytic Discoloration. ACS Appl. Nano Mater. 2023, 6, 14049–14062. 10.1021/acsanm.3c01935.

[ref47] Danilov M. O.; Dovbeshko G. I.; Rusetskyi I. A.; Bykov V. N.; Gnatyuk O. P.; Fomanyuk S. S.; Kolbasov G. Ya. Synthesis, properties and electrocatalytic application of g-C_3_N_4_ for oxygen electrodes of fuel cells. Nanocomposites 2023, 9, 1–9. 10.1080/20550324.2023.2169985.

[ref48] Peter L. M.; Walker A. B.; Bein T.; Hfnagel A. G.; Kondofersky I. Interpretation of photocurrent transients at semiconductor electrodes: Effects of band-edge unpinning. J. Electroanal. Chem. 2020, 872, 114234 10.1016/j.jelechem.2020.114234.

[ref49] Hojamberdiev M.; Mora-Hernandez J.; Vargas R.; Heppke E. M.; Yubuta K.; Yamakata A.; Kadirova Z.; Torres-Martínez L.; Teshima K.; Lerch M. Eliciting the contribution of TiN to photoelectrochemical performance enhancement of *Imma*-LaTiO_2_N at neutral pH. Mater. Today Energy 2022, 27, 101053 10.1016/j.mtener.2022.101053.

[ref50] Lu K.-Q.; Li Y.-H.; Tang Z.-R.; Xu Y.-J. Role of graphene oxide in heterogeneous photocatalysis. ACS Mater. Au 2021, 1, 37–54. 10.1021/acsmaterialsau.1c00022. 36855621 PMC9888622

[ref51] Monllor-Satoca D.; Díez-García M.; Lana-Villareal T.; Gómez R. Photoelectrocatalytic production of solar fuels with semiconductor oxides: materials, activity and modeling. Chem. Commun. 2020, 56, 12272–12289. 10.1039/D0CC04387G. 32960202

[ref52] Fujishima A.; Zhang X.; Tryk D. A. TiO_2_ photocatalysis and related surface phenomena. Surf. Sci. Rep. 2008, 63, 515–582. 10.1016/j.surfrep.2008.10.001.

[ref53] Koper M. T. M. Thermodynamic theory of multi-electron transfer reactions: Implications for electrocatalysis. J. Electrochem. Chem. 2011, 660, 254–260. 10.1016/j.jelechem.2010.10.004.

[ref54] Sambyal S.; Sharma R.; Mandyal P.; Balou S.; Gholami P.; Fang B.; Shandilya P.; Priye A. Advancement in two-dimensional carbonaceous nanomaterials for photocatalytic water detoxification and energy conversion. J. Environ. Chem. Eng. 2023, 11, 109517 10.1016/j.jece.2023.109517.

[ref55] Saleem Z.; Pervaiz E.; Yousaf M. U.; Niazi M. B. K. Two-Dimensional Materials and Composites as Potential Water Splitting Photocatalysts: A Review. Catalysts 2020, 10, 464 10.3390/catal10040464.

[ref56] Lim Y.; Lee D.-K.; Kim S. M.; Park W.; Cho S. Y.; Sim U. Low Dimensional Carbon-Based Catalysts for Efficient Photocatalytic and Photo/Electrochemical Water Splitting Reactions. Materials 2020, 13, 114 10.3390/ma13010114. PMC6982202 31881793

[ref57] Ge J.; Zhang Y.; Park S.-J. Recent Advances in Carbonaceous Photocatalysts with Enhanced Photocatalytic Performances: A Mini Review. Materials 2019, 12, 1916 10.3390/ma12121916. 31200594 PMC6631926

[ref58] Szunerits S.; Boukherroub R. Graphene-based nanomaterials in innovative electrochemistry. Curr. Opin. Electrochem. 2018, 10, 24–30. 10.1016/j.coelec.2018.03.016.

[ref59] Santos E.; Nazmutdinov R.; Schmickler W. Electron transfer at different electrode materials: Metals, semiconductors, and graphene. Curr. Opin. Electrochem. 2020, 19, 106–112. 10.1016/j.coelec.2019.11.003.

[ref60] Han Q.; Chen N.; Zhang J.; Qu L. Graphene/graphitic carbon nitride hybrids for catalysis. Mater. Horiz. 2017, 4, 832–850. 10.1039/C7MH00379J.

[ref61] Olatunde O. C.; Onwudiwe D. C. A Comparative Study of the Effect of Graphene Oxide, Graphitic Carbon Nitride, and Their Composite on the Photocatalytic Activity of Cu_3_SnS_4_. Catalysts 2022, 12, 14 10.3390/catal12010014.

[ref62] Pham T. A.; Ping Y.; Galli G. Modelling heterogeneous interfaces for solar water splitting. Nat. Mater. 2017, 16, 401–408. 10.1038/nmat4803. 28068314

[ref63] Liu L.; Tan S.; Horikawa T.; Do D. D.; Nicholson D.; Liu J. Water adsorption on carbon - A review. Adv. Colloid Interface Sci. 2017, 250, 64–78. 10.1016/j.cis.2017.10.002. 29129312

[ref64] Hojamberdiev M.; Piccirillo C.; Cai Y.; Kadirova Z. C.; Yubuta K.; Ruzimuradov O. ZnS-containing industrial waste: Antibacterial activity and effects of thermal treatment temperature and atmosphere on photocatalytic activity. J. Alloys Compd. 2019, 791, 971–982. 10.1016/j.jallcom.2019.03.368.

[ref65] Hojamberdiev M.; Kadirova Z. C.; Gonçalves R. V.; Yubuta K.; Matsushita M.; Teshima K.; Hasegawa M.; Okada K. Reduced graphene oxide-modified Bi_2_WO_6_/BiOI composite for the effective photocatalytic removal of organic pollutants and molecular modeling of adsorption. J. Mol. Liq. 2018, 268, 715–727. 10.1016/j.molliq.2018.07.110.

[ref66] Gracia J.; Kroll P. Corrugated layered heptazine-based carbon nitride: the lowest energy modifications of C_3_N_4_ ground state. J. Mater. Chem. 2009, 19, 3013–3019. 10.1039/b821568e.

[ref67] Carr A. J.; Lee S. E.; Uysal A. Ion and water adsorption to graphene and graphene oxide surfaces. Nanoscale 2023, 15, 14319–14337. 10.1039/D3NR02452K. 37561081

[ref68] Labille J.; Brant J.; Villiéras F.; Pelletier M.; Thill A.; Masion A.; Wiesner M.; Rose J.; Bottero J.-Y. Affinity of C_60_ Fullerenes with Water. Fullerenes, Nanotubes Carbon Nanostruct. 2006, 14, 307–314. 10.1080/15363830600665250.

[ref69] Rafii-Tabar H. Computational modelling of thermo-mechanical and transport properties of carbon nanotubes. Phys. Rep. 2004, 390, 235–452. 10.1016/j.physrep.2003.10.012.

[ref70] Wu H. Z.; Liu L. M.; Zhao S. J. The effect of water on the structural, electronic and photocatalytic properties of graphitic carbon nitride. Phys. Chem. Chem. Phys. 2014, 16, 3299–3304. 10.1039/c3cp54333a. 24413518

[ref71] Kesselman J. M.; Shreve G. A.; Hoffmann M. R.; Lewis N. S. Flux-Matching Conditions at TiO_2_ Photoelectrodes: Is Interfacial Electron Transfer to O_2_ Rate-Limiting in the TiO_2_-Catalyzed Photochemical Degradation of Organics?. J. Phys. Chem. 1994, 98, 13385–13395. 10.1021/j100101a044.

